# Evaluation of Commercially Available Diagnostic Tests for the Detection of Dengue Virus NS1 Antigen and Anti-Dengue Virus IgM Antibody

**DOI:** 10.1371/journal.pntd.0003171

**Published:** 2014-10-16

**Authors:** Elizabeth A. Hunsperger, Sutee Yoksan, Philippe Buchy, Vinh Chau Nguyen, Shamala Devi Sekaran, Delia A. Enria, Susana Vazquez, Elizabeth Cartozian, Jose L. Pelegrino, Harvey Artsob, Maria G. Guzman, Piero Olliaro, Julien Zwang, Martine Guillerm, Susie Kliks, Scott Halstead, Rosanna W. Peeling, Harold S. Margolis

**Affiliations:** 1 Dengue Branch, Centers for Diseases Control and Prevention, San Juan, Puerto Rico; 2 Center for Vaccine Development, Mahidol University, Bangkok, Thailand; 3 Virology, Institut Pasteur, Phnom Penh, Cambodia; 4 Hospital for Tropical Diseases, Cho Quan Hospital, Ho Chi Minh City, Vietnam; 5 Department of Medical Microbiology, University of Malaya, Kuala Lumpur, Malaysia; 6 Instituto Nacional Enfermedades Virales Humanas “Dr. Julio I. Maiztegui,” Pergamino, Argentina; 7 Instituto Medicina Tropical “Pedro Kouri,” Havana, Cuba; 8 Zoonotic Diseases and Special Pathogens, Public Health Agency of Canada, Winnipeg, Canada; 9 UNICEF/UNDP/World Bank/World Health Organization Special Programme for Research and Training in Tropical Diseases (TDR), Geneva, Switzerland; 10 Independent statistical consultant, Tak province, Thailand; 11 Pediatric Dengue Vaccine Initiative, Seoul, Korea; University of California, Davis, United States of America

## Abstract

Commercially available diagnostic test kits for detection of dengue virus (DENV) non-structural protein 1 (NS1) and anti-DENV IgM were evaluated for their sensitivity and specificity and other performance characteristics by a diagnostic laboratory network developed by World Health Organization (WHO), the UNICEF/UNDP/World Bank/WHO Special Programme for Research and Training in Tropical Diseases (TDR) and the Pediatric Dengue Vaccine Initiative (PDVI). Each network laboratory contributed characterized serum specimens for the panels used in the evaluation. Microplate enzyme-linked immunosorbent assay (ELISA) and rapid diagnostic test (RDT formats) were represented by the kits. Each ELISA was evaluated by 2 laboratories and RDTs were evaluated by at least 3 laboratories. The reference tests for IgM anti-DENV were laboratory developed assays produced by the Armed Forces Research Institute for Medical Science (AFRIMS) and the Centers for Disease Control and Prevention (CDC), and the NS1 reference test was reverse transcriptase polymerase chain reaction (RT-PCR). Results were analyzed to determine sensitivity, specificity, inter-laboratory and inter-reader agreement, lot-to-lot variation and ease-of-use. NS1 ELISA sensitivity was 60–75% and specificity 71–80%; NS1 RDT sensitivity was 38–71% and specificity 76–80%; the IgM anti-DENV RDTs sensitivity was 30–96%, with a specificity of 86–92%, and IgM anti-DENV ELISA sensitivity was 96–98% and specificity 78–91%. NS1 tests were generally more sensitive in specimens from the acute phase of dengue and in primary DENV infection, whereas IgM anti-DENV tests were less sensitive in secondary DENV infections. The reproducibility of the NS1 RDTs ranged from 92-99% and the IgM anti-DENV RDTs from 88–94%.

## Author Summary

Dengue virus (DENV) infection occurs throughout tropical and sub-tropical regions of the world where dengue is a major public health problem. Laboratory diagnosis of dengue with a single serum specimen obtained during the acute phase of the illness requires tests to detect IgM antibodies to DENV or the virus genome. A previous evaluation of available tests for IgM anti-DENV showed wide variability. The present study examined newly available commercial tests that detect the virus protein NS1, as well as new tests for IgM anti-DENV in microplate or rapid diagnostic test formats. This analytic study used specimens from laboratory confirmed dengue patients worldwide, which makes the results widely generalizable. The study found variability among the microplate ELISAs for both analytes but some tests performed with sensitivity and specificity acceptable for routine dengue diagnostics. The RDT's for both analytes had variable sensitivity that could be considered acceptable for routine clinical diagnostics. There is the need to maintain a network of dengue reference laboratories to conduct similar evaluations as additional dengue diagnostic tests become commercially available in order to guide the use for surveillance, clinical diagnosis and research.

## Introduction

Dengue is a major public health problem with more than 2.5 billion people at risk for DENV infection and an estimated 96 million cases occur annually in over 100 tropical and sub-tropical countries [1]–[3]. Infection with each of the four DENV (DENV serotypes 1–4) is capable of causing dengue fever as well as severe dengue. Currently there are no vaccines or drugs available to prevent or treat dengue. However, early laboratory diagnosis can ensure timely initiation of appropriate clinical management or anticipatory guidance in the outpatient setting. Accurate diagnosis of dengue is an important component of public health surveillance since clinical diagnosis does not differentiate dengue from other diseases that present with dengue-like signs and symptoms (e.g., malaria, leptospirosis, measles, influenza, Japanese encephalitis (JEV), West Nile fever (WNV), yellow fever virus (YFV)). Hence, there is the global need for accurate dengue diagnostics.

Timely and accurate laboratory diagnosis of dengue performed on a single serum specimen must rely on detection of DENV RNA or NS1 antigen during the period from fever onset until 5–6 days later, or detection of anti-DENV IgM beginning 3–5 days after fever onset until 6 weeks later [4]–[6]. DENV can be detected by virus isolation, molecular amplification of DENV RNA by RT-PCR and immunoassay to detect DENV NS1 antigen. As a diagnostic technique, virus isolation is not practical since it requires cell culture facilities, has a long turn-around time and has lower sensitivity compared to molecular or immunoassay methods [7]. In low resource settings, use of molecular tests is generally not feasible hence NS1 antigen detection may be the best option for DENV detection. The NS1 test appears to have adequate sensitivity and specificity when compared to RT-PCR and virus isolation across DENV serotypes; however, there are differences in NS1 sensitivity related to patient infection status (i.e., primary versus secondary DENV infection) [8]–[14]. Presently, the most widely used dengue diagnostic test remains the IgM capture anti-DENV (MAC) ELISA, which lacks adequate sensitivity and specificity in the acute phase of the illness, a time when most patients seek medical attention [15], [16].

Rapid diagnostic tests (RDTs) for dengue have become increasing available over the last 5 years because of the need for point-of-care diagnosis in resource limited settings. However, previously available RDTs for IgM anti-DENV did not have acceptable sensitivity compared to anti-DENV IgM microplate ELISAs which considerably limited their utility [17]. Although individual NS1 RDTs have been evaluated in a number of studies, robust estimates of their analytic performance have not been determined in head-to-head comparisons using specimen panels that replicate the diagnostic landscape of most dengue endemic areas.

To determine the analytic performance and reproducibility of commercially available NS1 antigen tests and newly available IgM anti-DENV RDTs and microplate ELISAs, a network of laboratories established by WHO/TDR and the PDVI developed specimen panels from dengue patients infected with all DENV serotypes from both the Asian and American continents, and with primary and secondary DENV infections. In addition, the evaluation included specimens from patients with other dengue-like illnesses and other conditions that can produce false-positive test results.

## Methods

### The Laboratory Network

The present study was conducted by the seven laboratories of the WHO/TDR/PDVI dengue diagnostic network that previously evaluated commercially available IgM anti-DENV diagnostic tests in 2007 [17]. Laboratories in Asia included: Centre for Vaccine Development, Mahidol University, Thailand; Hospital for Tropical Medicine, Cho Quan Hospital, Viet Nam; Institut Pasteur in Cambodia; and University of Malaya, Malaysia; and in the Americas: Dengue Branch, CDC, Puerto Rico; Instituto Medicina Tropical “Pedro Kouri”, Cuba; and Instituto Nacional Enfermedades Virales Humanas “Dr. Julio I. Maiztegui”, Argentina. The laboratories at CDC, Puerto Rico and Mahidol University, Thailand served as the reference laboratories and provided specimens for proficiency testing among the laboratories, assembled and validated the evaluation panels and distributed the test kits.

### Ethics Statement

This study was reviewed by the WHO Ethics Review Committee and considered exempt. All patient samples used for this study were de-linked from personal identifiers and renumbered with a study identifier.

### Study Initiatives

The mission of the Accessible Quality Assured Diagnostics program within the UNICEF/UNDP/World Bank/WHO Special Programme for Research and Training in Tropical Diseases (WHO/TDR) is to promote and facilitate development, evaluation and deployment of diagnostic tools for the control of the tropical diseases. PDVI was a product development partnership established to accelerate development, evaluation and introduction of dengue vaccines for children in endemic countries.

The previous study by the WHO/TDR/PDVI dengue diagnostic laboratory network resulted in recommendations to included three commercially available IgM anti-DENV ELISA tests in the WHO bulk procurement scheme (WHO report; [17]). Since this study, a number of tests for detection of NS1 antigen had become commercially available in addition to a number of new anti-DENV IgM tests. On 10–12 February 2009 the steering group and directors of the network laboratories met to plan the present study to evaluate the analytic performance and operational characteristics of newly available anti-DENV IgM and NS1 antigen detection tests in microplate ELISA and RDT formats for the diagnosis of dengue.

### Clinical Specimens

Specimen panels were created for anti-DENV IgM and NS1 testing (Table 1 and 2) based on the study design developed by the steering group with statistical consultation. Panels contained a sufficient number of true positive and negative specimens to give a confidence interval (CI) of +5% around the point estimates for test sensitivity and specificity [18]. Specimens were selected in a way that achieved geographic representation of the four DENV serotypes and were obtained from archived clinical samples at the participating laboratories and were submitted to the reference laboratories for assembly of the final panels. Case-specimens came from dengue patients that met the 1997 WHO dengue case classification and were laboratory confirmed by the presence of DENV detected by RT-PCR and/or virus isolation. All dengue cases included in the panels had paired, acute (≤5 days post onset of illness) and convalescent (6–14 days post onset of illness) specimens. However, the study design was not such that specimens for the acute and convalescent periods were allocated by specific days post onset of illness (see Supplemental Table S1), and analysis had to be performed for results from the aggregate acute and convalescent time periods. For certain categories in the challenge panels, serum specimens were purchased from SeraCare Diagnostics (West Bridgewater, MA).

**Table 1:** Characteristics of specimens from dengue patients in the panel to evaluate dengue virus (DENV) non-structural protein 1 (NS1) detection kits.

Panel	Region	Dengue infection*	Serotype	Acute¶	Convalescent#	Subtotal	Total
**NS1**	**Americas**	***Primary***	D1	4		4	
			D3	5		5	
			D4	1		1	
			***Subtotal***	***10***		***10***	**10**
		***Secondary***	D1	24		24	
			D2	25		25	
			D3	17		17	
			D4	9		9	
			***Subtotal***	***75***		***75***	**75**
	**Asia**	***Primary***	D1	3	5	8	
			D3	7	20	27	
			***Subtotal***	***10***	***25***	***35***	**35**
		***Secondary***	D1	4	12	16	
			D2	2	13	15	
			D3	4	22	26	
			D4	2	13	15	
			***Subtotal***	***12***	***60***	***72***	**72**
**Total**				**107**	**85**	**192**	**192**

*Primary  = 1 DENV infection, Secondary ≥2DENV infections.

¶ days post onset of fever  = 0–5.

# days post onset of fever  = 6–14.

**Table 2:** Characteristics of specimens from dengue patients included in the panel to evaluate anti-DENV IgM detection kits.

Panel	Region	Dengue infection*	Serotype	Acute¶	Convalescent#	Not defined	Subtotal	Total
**IgM**	**America**	***Secondary***	D1		29		29	
			D2		26		26	
			D3		17		17	
			D4		8		8	
			***Subtotal***		***80***		***80***	**80**
	**Asia**	***Primary***	D1	8			8	
			D2	3			3	
			D3	16			16	
			DF	1	3		4	
			DHF		4		4	
			***Subtotal***	***28***	***7***		***35***	**35**
		***Secondary***	D1	15			15	
			D2		10		10	
			D3	5	17		22	
			D4	4	19	4	27	
			Not defined	4	35		39	
			***Subtotal***	***28***	***81***	***4***	***113***	**113**
**Total**				**56**	**168**	**4**	**228**	**228**

DENV  =  dengue virus.

*Primary  = 1 DENV infection, Secondary ≥2 DENV infections.

¶ days post onset of fever  = 0–5.

# days post onset of fever  = 6–14.

The following epidemiological data were available for all case-specimens: date of onset of symptoms, date of sample collection, patient age, sex, summary of clinical history and diagnosis, travel history 12 months prior to the date of specimen collection, and vaccination history (i.e. YFV, JEV or other flavivirus vaccines). Case-patient specimens were classified as dengue fever (DF), dengue hemorrhagic fever (DHF), or dengue shock syndrome (DSS) based on data from clinical records using the 1997 WHO case definition.

Lipemic, hemolyzed or bacterially contaminated specimens were not included in the panels. Only specimens with ≤2 freeze-thaw cycles were included, all were heat-inactivated at 56°C for 30 minutes, lyophilized in aliquots, coded by the reference laboratories, assembled into the respective panels which were shipped to each evaluation laboratory. Written standard operating procedures were developed and used by participating laboratories to assure uniformity in sample handling, storage and testing. Because some commercial tests can be highly sensitive to storage and shipping conditions, data on transit time was recorded for later analysis.

### Testing Protocol

Letters requesting study participation were sent to 20 dengue diagnostic kit manufacturers; seven companies agreed to participate and provided the tests shown in Table 3 and supplemental Table S2 A–D. All kits were shipped by the manufacturers at ambient temperatures, checked for damage upon arrival and stored in the specified environment. RDT kits were shipped to only one of the reference laboratories where they were subsequently shipped to the evaluation laboratories under conditions specified by the manufacturer. Microplate ELISAs were only evaluated by the reference laboratories since the previous evaluation did not show significant inter-laboratory differences in results [17]. Delays in shipping or damage were noted and reported by the evaluation laboratories.

**Table 3:** Dengue virus NS1 antigen detection and IgM anti-dengue virus tests submitted for evaluation.

Test Type	Test Name	Company Name
**NS1 ELISAs**	Platelia Dengue NS1 Ag	Bio-Rad
	Dengue Early ELISA	Panbio/Alere
	Dengue NS1 Ag ELISA	Standard Diagnostics, Inc.
**NS1 RDTs**	Dengue NS1 Ag Strip	Bio-Rad
	OnSite Dengue Ag Rapid Test	CTK Biotech
	Dengue Early Rapid Test	Panbio/Alere
	SD BIOLINE Dengue Duo	Standard Diagnostics, Inc.
**IgM ELISA**	DENV-JEV MACE	Venture Technologies Sdn Bhd
**IgM RDTs**	Dengue IgG/IgM Rapid Test device	Abon Biopharma
	OnSite Dengue IgG/IgM Combo	CTK Biotech
	ImmunoComb II Dengue IgM & IgG Bispot	Orgenics/Iverness
	SD BIOLINE Dengue Duo	Standard Diagnostics, Inc.

Ten specimens known to be NS1 positive using the Platelia ELISA (BioRad, Lyon, France) were tested following lyophylization, heat inactivation and one freeze-thaw cycle and showed no change in sensitivity by any of these treatments (data not shown).

Specimens from each panel were tested in duplicate in the same analytical run to measure within-run precision; inter-run precision was evaluated by comparing results across evaluation laboratories. RDTs were tested in duplicates, with two readers evaluating each result independently. Inter-reader agreement was considered an important variable since RDTs required subjective judgment. All testing was conducted with strict adherence to the manufacturer's protocol described in the package insert. Each evaluation laboratory recorded testing data on standardized data sheets, which were submitted to WHO/TDR for analysis. Information was also collected from each performing technician regarding the ease-of-use of these products. This study was initiated in 2011 and completed within the same year.

### The Panels

#### DENV NS1 panel

This panel consisted of a total of 390 dengue patient serum specimens in 3 sub-panels: 192 dengue patients who were DENV positive by virus isolation and/or RT-PCR for DENV RNA, 146 DENV RNA or culture negatives and 52 challenge specimens from other illnesses or conditions that may result in false positive results to determine the specificity of these tests (Tables 1 and 4). DENV RNA or culture positive serum specimens were collected 0–5 days post onset of fever (DPO). All dengue cases had paired acute (DPO 0–5) and convalescent (DPO 6–14) specimens.

**Table 4:** Characteristics of DENV negative specimens and challenge specimens for the evaluation of NS1 and anti-DENV IgM tests.*

	NS1			IgM		
	America	Asia	Total	America	Asia	Total
**Negatives**						
Anti-DENV IgM and RT-PCR negative*	89	57	**146**	**86**	**69**	**155**
**Systemic conditions**						
Rheumatoid Arthritis	10			9		
Systemic Lupus Erythematosus	2			2		
***Total***	***12***		***12***	***11***		***11***
**Challenge Panel** **						
**Febrile illnesses**						
IgG anti Lyme positive	10			10		
IgM anti Hantavirus positive	6			9		
IgM-IgG anti Hantavirus positive	1			1		
IgG anti Hantavirus positive	1			2		
Chikungunya					10	
Scrub typhus					12	
Leptospirosis				12	7	
Malaria					30	
***Total***	***18***	***0***	***18***	***34***	***59***	***93***
**Related flavivirus**						
IgM anti West Nile positive	10			10		
IgM anti Yellow Fever positive	3				3	
IgG anti Yellow Fever positive	2			4		
Flavivirus	1			4	10	
IgM-IgG anti St Louis Encephalitis positive	3					
IgG anti St Louis Encephalitis positive	1					
Remote dengue (anti-DENV IgG positive)	2					
***Total***	***22***	***0***	***22***	***18***	***13***	***31***
***Other***						
Pregnancy					***10***	***10***
**Subtotal Challenge Panel**	**141**	**57**		**149**	**151**	
**Total**			**198**			**300**

NS1  =  non-structural protein 1, DENV  =  dengue virus.

*specimens from persons living in dengue non-endemic areas and negative for IgM and IgG antibody and reverse transcriptase polymerase chain reaction (RT-PCR) to dengue virus (DENV).

**specimens from systemic conditions, other febrile illnesses, related flavivirus infections and past DENV infections.

Network laboratories tested a limit of detection panel to ensure comparable sensitivity of their RT-PCR assay as a reference standard. The methods of RNA extraction and RT-PCR used in the network laboratories included: nested RT-PCR by Lanciotti *et al*., 1992 [18], real-time RT-PCR methods developed by Laue et al., 1999 [19] Kong et al., 2006 [20]and Chien et al., 2006 [21]. Network laboratories tested a panel of serially diluted cell culture-derived DENV RNA to determine the limit of detection of their RT-PCR assays and ensure comparable testing sensitivity. For virus isolation, cell culture isolation was performed using an accepted reference method [22].

DENV positive specimens included patients with primary and secondary DENV infections and represented all 4 DENV serotypes. Primary and secondary infections were distinguished according to criteria established by WHO using hemagglutination-inhibition assay described by Clark and Casals, 1958 adapted to 96-well microtiter plate [23], [24]. This approach was adapted to IgG ELISA where a primary DENV infection was defined as evidence of first exposure to DENV based on anti-DENV IgG seroconversion between and acute and convalescent specimen, and a secondary DENV infection was defined as evidence of more than one DENV infection as determined by anti-DENV IgG positive titer during the acute phase of disease [25].

This panel also included paired, acute and convalescent specimens from patients who were DENV RNA and/or virus isolation positive and anti-DENV IgM negative in the acute phase specimens but DENV RNA and/or virus isolation negative and anti-DENV IgM positive in the convalescent specimens. These specimens were used to determine the duration of NS1 antigen detection over the course of the illness. The NS1 panel was used to test microtiter ELISAs and RDTs. All tests were performed in duplicates including the RDTs. Since each RDT represents a single test the number of specimens in the table are including the duplicate and is exactly twice as many specimens number as the ELISA tests.

#### Negative NS1 panel

The subpanels of DENV negatives and challenge specimens were obtained from normal, healthy persons living in dengue non-endemic areas and were negative for DENV by RT-PCR and/or virus isolation, and IgM and IgG anti-DENV negative. The challenge specimens included individuals with non-DENV flavivirus illnesses, febrile illnesses of other etiologies, or systemic conditions known to give rise to false positive results in immunoassays (Table 4) and some were purchased from a commercial source (SeraCare, West Bridgewater, MA).

#### Anti-DENV IgM panel

This panel consisted of 3 subpanels with 527 patient serum specimens: 228 anti-DENV IgM positive paired specimens from patients with dengue (Table 2) and 155 DENV IgM negative and 144 challenge specimens (Table 4). All specimens were tested by the solid-phase anti-DENV IgM MAC-ELISA used by CDC or AFRIMS [26], [27]; a positive result by either test was considered the reference value. All samples were from paired specimens as previously defined for the NS1 panel. Positive specimens were selected based on optical density (OD) in the respective tests, and weighted towards low and medium ODs to increase the panel's power to evaluate test sensitivity.

#### Anti-DENV IgM negative panel

DENV IgM negative specimens were obtained from healthy individuals living in dengue non-endemic areas and were negative for DENV RNA and/or virus isolation, and IgM and IgG anti-DENV. Challenge specimens were obtained from patients with febrile illness of other etiologies, non-DENV flavivirus illnesses or systemic conditions known to give rise to false positive results in immunoassays (Table 4). Challenge specimens were solid-phase anti-DENV IgM negative and some were purchased from a commercial source (SeraCare, West Bridgewater, MA).

### Statistical Analysis

Sensitivity and specificity were assessed against reference test by analyte evaluated (NS1 or IgM). Kappa is a measure of the degree of non-random agreement between observers or measurements of the same categorical variable. Agreement is considered as good if kappa is between 0.60 and 0.80, and very good if greater than 0.80.

The McNemar paired test was used to compare the difference of agreement between operators. Proportions were compared using the chi-square test or the Fischer exact test, whichever was appropriate. Confidence intervals were set at 95% (CI_95_). P-values lower than 0.05 were considered as statistically significant. The statistical program used was STATA (version 11, Stata Corp.).

## Results

### DENV NS1 Microplate ELISAs

The sensitivities of the NS1 ELISAs in specimens from dengue patients during the acute phase of illness ranged from 60–75% (Table 5). Based on the overall results, test performance in order of most sensitive to least sensitive was: Panbio E, Standard Diagnostics and BioRad Platelia.

**Table 5:** Sensitivity of DENV NS1 antigen compared to RT-PCR and/or virus isolation in acute and positive rate of the convalescent specimens.

			Acute			Convalescent		
	Tests		Positive	Total n = 107	Sensitivity* (CI_95_)	Positive	Total n = 85	Sensitivity¶ (CI95)
NS1	ELISA	Bio-Rad	64	106¶	60% (51–70)	24	83¶	29% (19–39)
		Panbio	78	104¶	75% (67–83)	16	84¶	19% (11–27)
		SD	74	105¶	70% (62–79)	26	85	31% (21–40)
	RT	Bio-Rad	104	n = 214 199¶	52% (45–59)	32	n = 170 170	19% (13–25)
		CTK	50	125¶	40% (31–49)	33	170	19% (13–35)
		Panbio	119	197¶	60% (54–67)	21	170	12% (7–17)
		SD Duo NS1	115	195¶	59% (52–66)	100	170	59% (51–66)

			Primary			Secondary		
	Tests		Positive	Total n = 45	Sensitivity* (CI_95_)	Positive	Total n = 147	Sensitivity¶ (CI_95_)
NS1	ELISA	Bio-Rad	26	43¶	60% (46–75)	62	147	42% (34–50)
		Panbio	28	43¶	65% (51–79)	66	146¶	45% (37–53)
		SD	33	44¶	75% (62–88)	67	147	46% (38–54)
	RT	Bio-Rad	46	n = 90¶ 78¶	59% (48–70)	90	n = 294¶ 293¶	31%(25–36)
		CTK	37	78¶	47% (36–59)	46	218¶	21% (16–27)
		Panbio	30	78¶	38% (28–49)	110	291¶	38% (32–43)
		SD duo NS1	55	78¶	71% (60–81)	160	289¶	55% (50–61)

Sensitivity of dengue NS1 antigen in primary and secondary DENV infection status.

*Comparison to RT-PCR DENV positive samples,

¶ Comparison to IgM seroconversion

¶ Number of samples tested differed to total number due to either duplicates for RDTs, invalid test or equivocal result.

Acute is days post onset of fever (DPO) of 0–5 days, convalescent is DPO = 6–14.

Primary = 1 DENV infection, Secondary ≥2 DENV infections.

CI95 = 95% confidence interval.

When specimens collected at DPO 6–14 were tested, the test with the highest positive rate was Standard Diagnostics 31% (CI_95_ 21–40 p = <.001) and the lowest positive rate was Panbio E 19% (CI_95_ 11–27; p<.001), (Table 5). All of these rates were statistically different from those observed in the acute phase specimens (DPO 0–5) and were statistically different between tests.

Analysis by primary versus secondary DENV infection showed that sensitivity was significantly affected for all tests. For persons with a primary DENV infection, the test with the highest sensitivity was Standard Diagnostics 75% (CI_95_ 62–88), and the least sensitive was Bio-Rad Platelia 60% (CI_95_ 46–75); these results were significantly higher than observed among dengue cases with secondary infections: Standard Diagnostics 46% (CI_95_ 38–54; p = .001), BioRad Platelia 42% (CI_95_ 34–50; p = .03). This analysis included both acute and convalescent specimens (Table 5 and 6).

**Table 6:** Sensitivity of anti-DENV IgM compared to IgM reference test in acute and convalescent samples.

			Acute			Convalescent		
		Tests	Positive	Total n = 56	Sensitivity* (CI_95_)	Positive	Total n = 168	Sensitivity* (CI_95_)
IgM	ELISA	Venture	55	56	98% (95–100)	160	165¶	97% (94–100)
	RT	Abon	67	n = 112 107¶	63% (53–72)	187	n = 336 334¶	56% (51–61)
		CTK	51	112	46% (36–55)	178	334¶	53% (48–59)
		Orgenic	100	112	95% (90–99)	250	255¶	82% (78–87)
		SD duo IgM	106	112	89% (84–95)	210	255¶	98% (96–100)

			Primary			Secondary		
		Tests	Positive	Total n = 35	Sensitivity* (CI_95_)	Positive	Total n = 193	Sensitivity* (CI_95_)
IgM	ELISA	Venture	34	35	97% (92–100)	184	191¶	96% (94–99)
	RT	Abon	49	N = 70¶ 65¶	75% (65–86)	212	N = 386¶ 386	55% (50–60)
		CTK	21	70	30% (19–41)	214	386	55% (50–60)
		Orgenic	59	70	84% (76–93)	298	306¶	97% (96–99)
		SD duo IgM	67	70	96% (91–100)	256	306¶	84% (80–88)

Sensitivity of dengue anti-DENV IgM compared to IgM reference test in primary and secondary DENV infection status.

*Comparison to anti-DENV IgM reference positive samples.

¶ Number of samples tested differed to total number due to either duplicates for RDTs, invalid test or equivocal result.

Acute is days post onset of fever (DPO) of 0–5 days, Convalescent is DPO = 6–14.

Primary  = 1 DENV infection, Secondary ≥2 DENV infections.

CI95 = 95% confidence interval.

**Figure 1:**
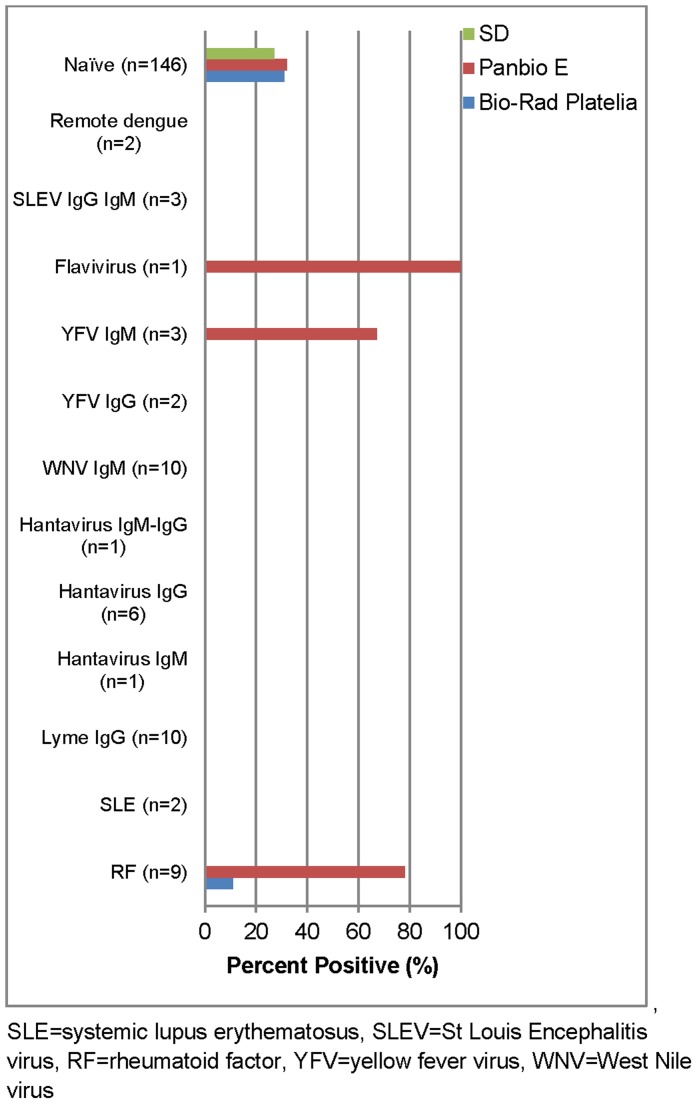
False positive rate of DENV NS1 ELISAs in DENV negatives and challenge panel specimens.

The specificity of these kits, determined with the DENV negative and challenge specimen panels, is shown in Figure 1 and ranged from 71–80%. Few specimens gave a false positive result in the challenge panel; rheumatoid factor (RF) caused a false positive result in 78% and 11% of the Panbio E and BioRad Platelia NS1 ELISA, respectively and positive specimens for YFV and non-DENV flavivirus infections were reactive in the Panbio NS1 ELISA (Figure 1).

### DENV NS1 RDTs

The sensitivities of NS1 RDTs in the acute phase specimens (DPO 0–5) ranged from 40 to 59%: Panbio 60% (CI_95_ 54–67) was most sensitive and CTK Biotech 40% (CI_95_ 31–49) the least (Table 5). Among specimens collected in the convalescent phase of dengue (DPO 6–14), the highest test positive rate was for the SD BIOLINE Dengue Duo 59% (CI_95_ 51–66); and the lowest test positive was the Panbio 12% (CI_95_ 7–17); With the exception of SD BIOLINE Dengue Duo RDT, all of these were lower than the rates observed in the acute phase specimens.

Analysis by primary versus secondary DENV infection showed that most of the tests performed best among specimens from patients with primary infections: SD BIOLINE Dengue Duo 71%, (CI_95_ 60–81) vs. 55% (CI_95_ 50–61), (p = .016); Bio–Rad RDT 59% (CI_95_ 48–70) vs. 31% (CI_95_ 25–36), (p<.001); CTK Biotech 47% (CI_95_ 36–59) vs. 21% (CI_95_ 16–27), (p<.001), and Panbio 38% (CI_95_ 28–49) vs. 38% (CI_95_ 32–43) (p = .915) (Table 5).

**Figure 2:**
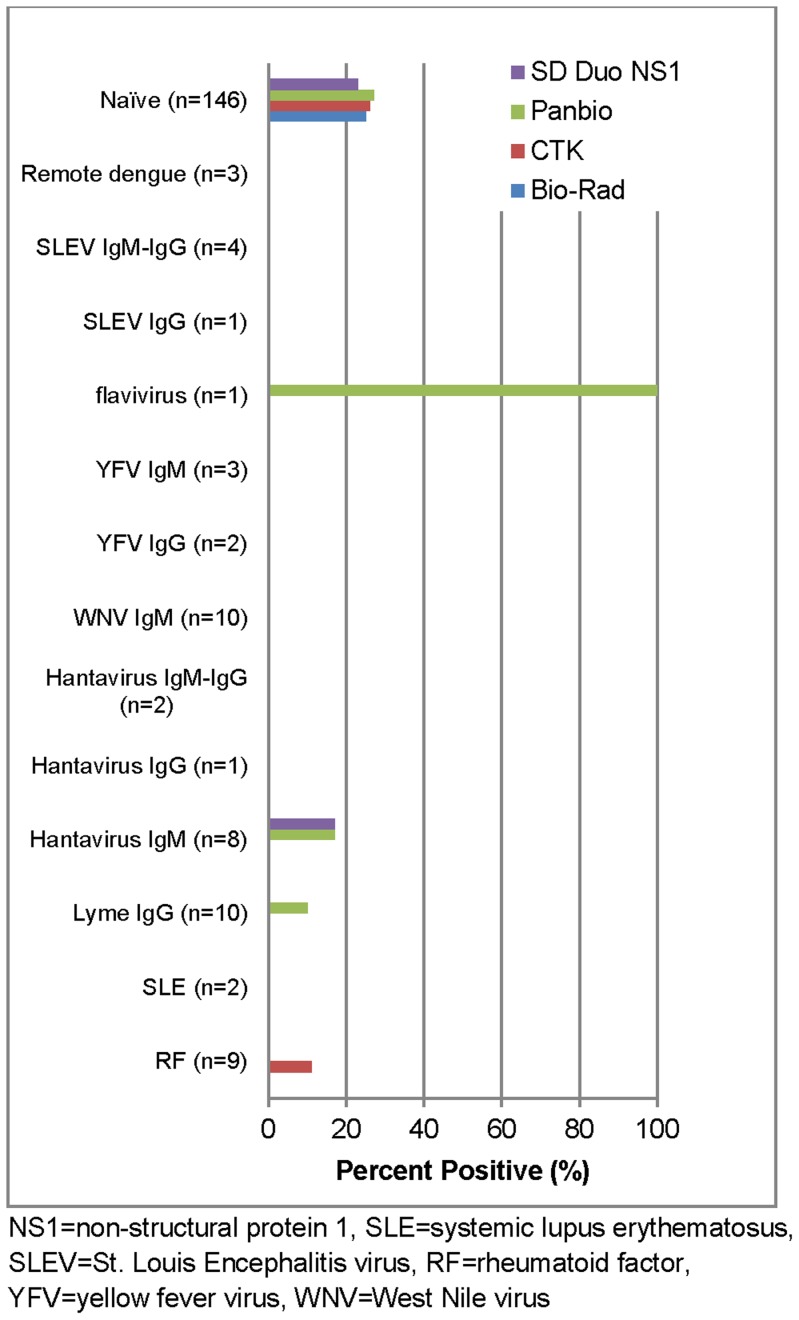
False positive rate DENV NS1 rapid diagnostic tests in DENV negatives and challenge panel specimens.

All four RDTs had similar specificities in the DENV negative specimens (76–80%) (Figure 2). Against the challenge specimens, the CTK Biotech test had reactivity against RF and the SD BIOLINE Dengue Duo and Panbio tests had reactivity against Hantavirus. All the NS1 RDTs had very good agreement between operators with kappa values ranging from 0.84–0.99 (Table 7).

**Table 7:** Test agreement between operators.

Detection type	Tests*	N	Agreement	Kappa		
				Value	CI_95_	p**
**NS1**	Bio-Rad RT	661	99%	0.99	0.97–1.00	1.000
	CTK RTNS1	539	98%	0.94	0.91–0.97	0.388
	Panbio RT	660	97%	0.93	0.91–0.96	0.019
	SD RT duo	654	92%	0.84	0.80–0.88	0.322
**IgM**	Abon RT	917	91%	0.79	0.74–0.83	0.590
	CTK RTIgM	887	94%	0.88	0.84–0.91	0.322
	Orgenics RT	802	88%	0.76	0.71–0.80	0.417
	SD RT duo	805	91%	0.81	0.77–0.85	1.000

Each test (anti-DENV IgM rapid test (RT) and the DENV NS1 antigen RT) was read and recorded independently by two operators. The agreement between the operators was reflected as kappa values.

*Each test was read and recorded independently by two operators.

**McNemar test.

### Anti-DENV IgM ELISA

**Figure 3:**
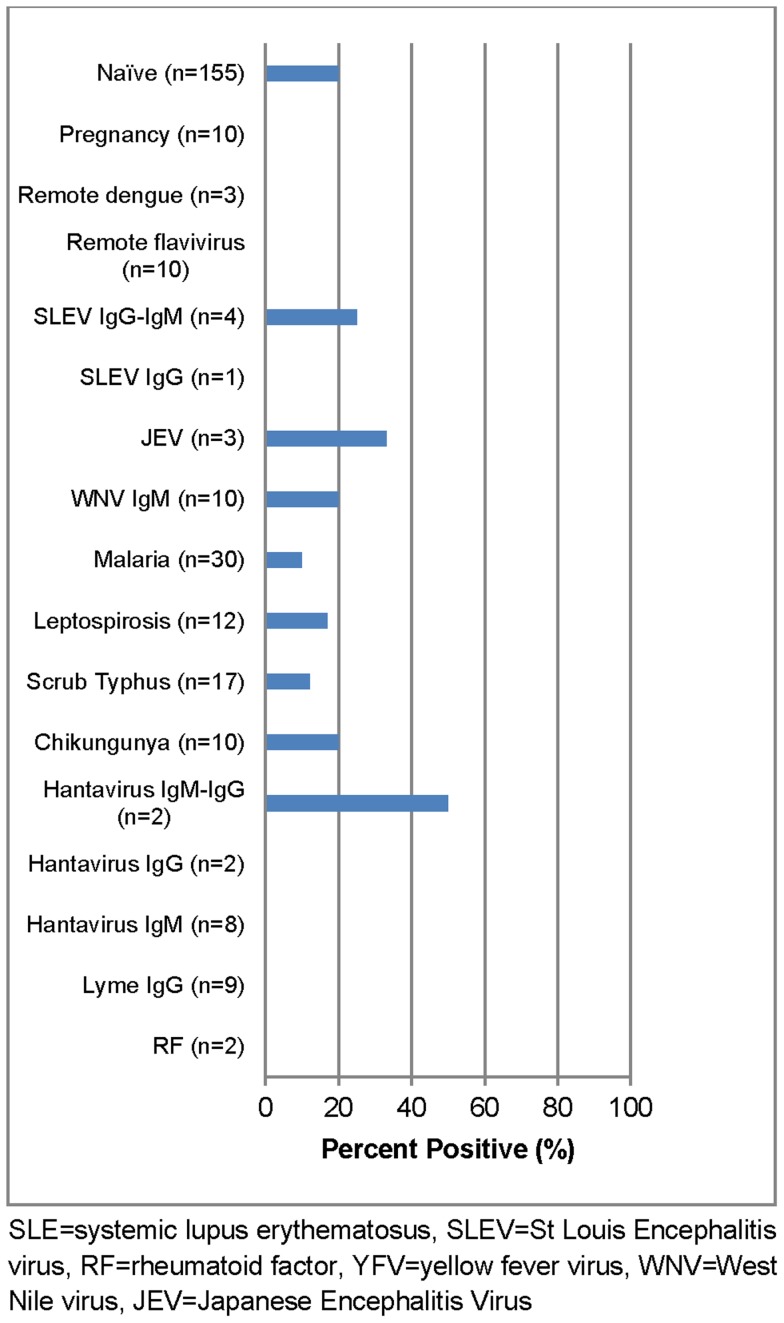
False positive rate of anti-DENV IgM ELISA (Venture Technologies Sdn Bhd) in DENV negatives and challenge panel specimens.

Only Venture E submitted a test kit for evaluation which showed an overall sensitivity of 96% (CI_95_ 94–99). Comparison of acute versus convalescent specimens demonstrated 98% (CI_95_ 95–100) sensitivity in acute phase specimens and 97% (CI_95_ 94–100) sensitivity in convalescent specimens. When analyzed according to DENV infection status, the sensitivity was 97% (CI_95_ 92–100) in primary infections compared to 96% (CI_95_ 94–99) in secondary DENV infections (Table 6). The overall specificity was 84% (CI_95_ 80–89) against the DENV negative panel. False positives reactions were observed against other flavivirus infections - St Louis Encephalitis virus (SLEV), Japanese Encephalitis virus (JEV) and West Nile virus (WNV), as well as Chikungunya virus (CHIKV) and Hantavirus. False positives rates were also observed for malaria, leptospirosis and scrub typhus (Figure 3).

### Anti-DENV IgM RDTs

The overall sensitivities of the IgM RDTs ranged from 52–95%. In the acute phase panel, the most sensitive test was Orgenics 95% (CI_95_ 90–99), and the least sensitive was CTK Biotech 46% (CI_95_ 36–55) (Table 6). In the convalescent panel sensitivities were higher than those observed in the acute panel for the following tests: CTK Biotech  = 53% (CI_95_ 48–59; p = .155) and SD BIOLINE Dengue Duo IgM = 98% (CI_95_ 96–100; p = .002). The following tests had lower sensitivities in the convalescent panel when compared to the acute panel: Orgenics 82% (CI_95_ 78–87; p<.001) and Abon 56% (CI_95_ 51–61; p = .227).

When evaluated based on DENV infection status, the SD BIOLINE Dengue Duo had a sensitivity of 96% (CI_95_ 91–100) in primary infections compared to 84% (CI_95_ 80–88) for secondary infections (p = .009) and the Abon test had a sensitivity of 75% (CI_95_ 65–86) for primary infections compared to 55% (CI_95_ 50–60) for secondary infections (p = .002). RDTs with statistically significant increase in sensitivity in secondary infections, included: Orgenics 84% (CI_95_ 76–93) compared to 97% (CI_95_ 96–99) (p<.001) and CTK Biotech 30% (CI_95_ 19–41) compared to 55% (CI_95_ 50–60) (p = <.001) for primary and secondary, respectively (Table 6).

**Figure 4:**
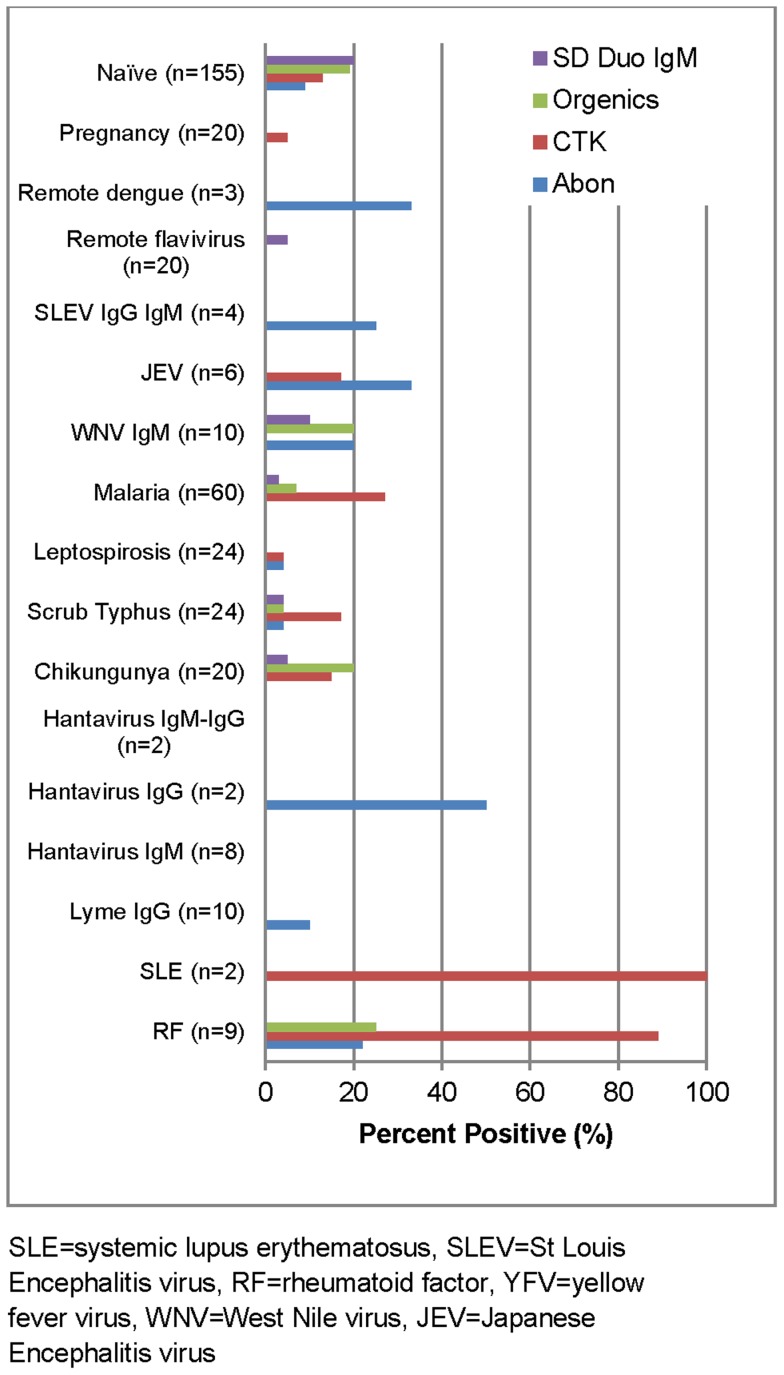
False positive rate of anti-DENV IgM rapid diagnostic tests in DENV negatives and challenge panel specimens.

The overall specificity of these RDTs ranged from 86–92%, with the highest false positive rate in the CTK Biotech test for RF and systemic lupus erythematosus (Figure 4). The kappa values for reader-to-reader variability ranged from 0.76–0.99 (Table 7).

## Discussion

This laboratory-based, analytic evaluation of commercially available dengue diagnostic tests for NS1antigen and anti-DENV IgM used a sufficient number of specimens to yield confidence intervals of +5% around the point estimates of test sensitivity and specificity [28]. Test sensitivity was determined in the acute and convalescent phases of dengue illness where a reference test was available (i.e., IgM anti-DENV) and in patients with primary and secondary DENV infections. The goal of this study was to provide reliable information on product performance to those performing dengue diagnostics, as well as to the test manufacturers.

DENV NS1 antigen is present in the acute phase of dengue with peak detection at DPO 3 followed by a rapid decline through the convalescent phase (DPO>5) [29]. The 3 microplate ELISAs evaluated in our study showed similar findings. During the convalescent phase of illness, previous studies have shown test positivity ranging from 22% to 40% among dengue cases defined by a positive IgM anti-DENV seroconversion between acute and convalescent specimens [12], [30], [31], a finding similar to ours.

Since NS1 is a viral antigen, the only available reference standard has been the presence of DENV as determined by RT-PCR. However, viral antigens may persist in serum longer than viral nucleic acid detected by molecular amplification or virus detected by cell culture. Thus, the interpretation of NS1 ELISA and RDT results obtained from specimens in the convalescent panel is not straightforward. All specimens in our panel were anti-DENV IgM positive but negative for DENV by RT-PCR or virus isolation. By strict definition, we were not able to determine the sensitivity of these tests in convalescent phase specimens because there was no reference standard for comparison. One interpretation is these are false-positive results since the specimens were DENV RNA negative. However, it has been shown in longitudinal studies of dengue patients that NS1 antigen remains positive after DENV RNA amplicons disappear [12], [30], [31] probably because of longer half-life of NS1 protein. The 19–30% test positivity in the convalescent panel could represent detection of residual NS1 antigen although some of these could also be false positive results. Until a protein based reference standard for NS1 is developed or a confirmatory test for NS1 test-reactivity is developed, study designs that follow patients sequentially during their illness are required to determine the diagnostic utility of this test in the early convalescent phase of dengue.

Our study showed higher sensitivity in primary than secondary DENV infections for both NS1 ELISAs and RDTs, with the exception of the Panbio RDT which had equal sensitivity (Table 5). The analysis included specimens from both acute and convalescent phase. This difference in NS1 detection has been observed previously [12], [30], [31] and may be explained by presence of anti-NS1 IgG, which occurs most frequently in secondary DENV infections, and may mask antigen detection by immune complex formation. Higher NS1 ELISA sensitivity has been reported for primary DENV infections not containing NS1 immune complexes compared to secondary DENV infections with complexes.

Although generally the NS1 RDTs had reduced sensitivities compared to the NS1 ELISAs, they had similar specificities, and the RDT's good agreement (>.80) between operators. However, there were still differences noted between laboratories as observed previously in the evaluation of the IgM anti-DENV RDTs [17].

As might be expected, there were significant differences in sensitivity between acute and convalescent specimens for the IgM anti-DENV RDTs because anti-DENV IgM peaks at DPO 10, except for the Abon RDT which had essentially similar sensitivity in the both periods. Anti-DENV IgM titers have been shown to be higher in primary compared to secondary infections, with as many as 20% of dengue cases with secondary infection having undetectable levels of anti-DENV IgM in convalescent specimens [16]. When examined by DENV infection status, the present evaluation showed that two of the tests, CTK Biotech and Orgenics, had significantly higher sensitivity in secondary compared to primary infections.

The utility of using both IgM anti-DENV and NS1 antigen for dengue diagnostic testing has been evaluated in recent studies, suggesting an added diagnostic benefit to this testing combination. [14], [32] Measuring both analytes during the early course of the illness (DPO 0–8) expands the diagnostic window of opportunity since NS1 detection is best detected during DPO 0–5 and anti-DENV IgM is best detected during DPO 5–14.

This study had several limitations as well as strengths. Test performance was compared to the evaluation panels, which were only characterized by the two reference laboratories using the reference methods. Additionally, the NS1 and IgM anti-DENV test sensitivity could not be evaluated by DENV serotype because of an insufficient number of specimens to allow for this sub-analysis. In addition, the small number of samples in some of the challenge panel categories did not provide strong point and confidence interval estimates similar to those derived for the DENV positive panels, and only identified potential issues. Lastly, the infectious agents represented in the challenge panels may not be of epidemiologic importance in all populations where dengue occurs. The convalescent specimens used for the NS1 evaluation were not characterized with the same reference tests as the acute specimens and no further testing was performed to determine if a positive result was a true or false positive. The strength of the study was that a high proportion of specimens in the panels were from persons with secondary DENV infections which, reflects the situation in most dengue endemic countries. In addition, most tests tended to perform better for primary DENV infections, which would mitigate any concerns about test performance in patients with this infection status (e.g., travelers from non-endemic countries).

Results from this evaluation have been provided to the manufacturers and WHO member states and provide benchmarks for the evaluation, procurement and use of dengue diagnostic tests at the country or regional level. This study indicates that most of the NS1 microplate ELISAs performed adequately for routine, clinical diagnostic use in dengue endemic countries. However, the clinical and epidemiologic consequences of the lower sensitivity of NS1 tests compared to DENV RNA detection should be considered when using these tests for routine use.

The microplate anti-DENV IgM ELISA continues to show good performance and this evaluation indicates that the one test evaluated had good sensitivity in the early phase of the illness, an area that has been problematic in the past. Whether these tests should be combined with NS1 microplate ELISAs for routine testing or in a testing algorithm based on when a patient presents for clinical care, needs to be evaluated in large-scale clinical studies.

Unfortunately the RDTs for both NS1 and anti-DENV IgM continue to have relatively poor performance profiles; a problem for resource poor countries. Since the combination NS1/anti-DENV IgM RDTs was only evaluated for each analyte individually, no statement can be made regarding its performance as a combination test. This study did not address the issue of how RDTs, with their lower sensitivity, might be used in resource poor areas. Even with their lower sensitivity but comparable specificities compared the ELISAs, they could be used to identify dengue outbreaks. However, large studies are needed to determine the most effective way to use them for case management, disease surveillance or whether their present shortcomings can be overcome by a combination testing algorithm.

## Supporting Information

Checklist S1 STARD checklist and STARD flowchart.
(DOC)

Table S1 Number of dengue virus (DENV) positive specimens in the non-structural protein 1 (NS1) panel (Table 1) by infection status (primary vs. secondary DENV infection) and days post onset of fever (DPO).
(DOCX)

Table S2 Commercial test characteristics of: A) NS1 ELISAs B) NS1 rapid diagnostic tests (RDTs) C) anti-DENV IgM ELISA and D) anti-DENV IgM RDTs.
(DOCX)
