# Supplementary material for: Evaluation of Commercially Available Diagnostic Tests for the Detection of Dengue Virus NS1 Antigen and Anti-Dengue Virus IgM Antibody
Source: PLoS Negl Trop Dis. 2014 Oct 16;8(10):e3171. doi: 10.1371/journal.pntd.0003171 (PMC4199549; doi:10.1371/journal.pntd.0003171)
Supplement: Table S1 — Number of dengue virus (DENV) positive specimens in the non-structural protein 1 (NS1) panel (Table 1) by infection status (primary vs. secondary DENV infection) and days post onset of fever (DPO). (DOCX) [file pntd.0003171.s002.docx]

**Supplemental Table 1.** Number of dengue virus (DENV) positive specimens in the non-structural protein 1 (NS1) panel (Table 1) by infection status (primary vs. secondary DENV infection) and days post onset of fever (DPO)

|  | **Days post onset of fever (DPO)*** | | | | | | | | | | | | | **Total** |
| --- | --- | --- | --- | --- | --- | --- | --- | --- | --- | --- | --- | --- | --- | --- |
|  | **0** | **1** | **2** | **3** | **4** | **5** | **6** | **7** | **8** | **9** | **10** | **11** | **12** |  |
| Number of DENV Positives from Primary Infections^¶^ | 1 | 7 | 0 | 3 | 3 | 6 | 8 | 5 | 4 | 2 | 1 | 2 | 3 | **45** |
| Number of DENV Positives from Secondary Infections^¶^ | 2 | 7 | 9 | 22 | 30 | 17 | 6 | 8 | 6 | 8 | 15 | 10 | 7 | **147** |
|  | **Acute Specimens=107** | | | | | | **Convalescent Specimens=85** | | | | | | | |
| **Total Specimens** | **192** | | | | | | | | | | | | | |

**DPO=0-5 were positive for DENV by RT-PCR and/or viral isolation and DPO=6-12 were from paired specimens in which the acute specimen was positive by RT-PCR and/or viral isolation and the convalescent specimen was anti-DENV IgM positive*

^¶^ *Primary=1 DENV infection, Secondary ≥2DENV infections*
