# Supplementary material for: Evaluation of Commercially Available Diagnostic Tests for the Detection of Dengue Virus NS1 Antigen and Anti-Dengue Virus IgM Antibody
Source: PLoS Negl Trop Dis. 2014 Oct 16;8(10):e3171. doi: 10.1371/journal.pntd.0003171 (PMC4199549; doi:10.1371/journal.pntd.0003171)
Supplement: Table S2 — Commercial test characteristics of: A) NS1 ELISAs B) NS1 rapid diagnostic tests (RDTs) C) anti-DENV IgM ELISA and D) anti-DENV IgM RDTs. (DOCX) [file pntd.0003171.s003.docx]

**Supplemental Table 2.** Commercial test characteristics of: A) NS1 ELISAs B) NS1 rapid diagnostic tests (RDTs) C) anti-DENV IgM ELISA and D) anti-DENV IgM RDTs

**A)**

**B)**

**C)**

**D)**
